# Evaluation of the Relative Validity of the Short Diet Questionnaire for Assessing Usual Consumption Frequencies of Selected Nutrients and Foods

**DOI:** 10.3390/nu7085282

**Published:** 2015-08-04

**Authors:** Bryna Shatenstein, Hélène Payette

**Affiliations:** 1 Centre de Recherche, Institut Universitaire de Gériatrie de Montréal, CIUSSS du Centre-est-de-l’Île-de-Montréal, Montréal, QC H3W 1W5, Canada; 2 Département de Nutrition, Université de Montréal, Montréal, QC H3T 1A8, Canada; 3 Centre de Recherche sur le Vieillissement, CIUSSS de l’Estrie-CHUS Sherbrooke, Sherbrooke, QC J1J 3H5, Canada; E-Mail: helene.payette@usherbrooke.ca; 4 Département des Sciences de la Santé Communautaire, Faculté de Médecine et des Sciences de la Santé, Université de Sherbrooke, Sherbrooke, QC J1H 5N4, Canada

**Keywords:** dietary assessment, brief tools, food frequency questionnaires, validation study, aging

## Abstract

A 36-item Short Diet Questionnaire (SDQ) was developed to assess usual consumption frequencies of foods providing fats, fibre, calcium, vitamin D, in addition to fruits and vegetables. It was pretested among 30 community-dwelling participants from the Québec Longitudinal Study on Nutrition and Successful Aging, “NuAge” (*n* = 1793, 52.4% women), recruited in three age groups (70 ± 2 years; 75 ± 2 years; 80 ± 2 years). Following revision, the SDQ was administered to 527 NuAge participants (55% female), distributed among the three age groups, both sexes and languages (French, English) prior to the second of three non-consecutive 24 h diet recalls (24HR) and validated relative to the mean of three 24HR. Full data were available for 396 participants. Most SDQ nutrients and fruit and vegetable servings were lower than 24HR estimates (*p* < 0.05) except calcium, vitamin D, and saturated and *trans* fats. Spearman correlations between the SDQ and 24HR were modest and significant (*p* < 0.01), ranging from 0.19 (cholesterol) to 0.45 (fruits and vegetables). Cross-classification into quartiles showed 33% of items were jointly classified into identical quartiles of the distribution, 73% into identical and contiguous quartiles, and only 7% were frankly misclassified. The SDQ is a reasonably accurate, rapid approach for ranking usual frequencies of selected nutrients and foods. Further testing is needed in a broader age range.

## 1. Introduction

The Canadian Longitudinal Study on Aging (CLSA) is a national longitudinal study that will follow 50,000 men and women aged 45 to 85 years (y) at recruitment over a twenty-year period. The goal of the CLSA is to better understand the aging process and its determinants through the collection of information on biological, medical, psychological, social, lifestyle and economic aspects of people’s lives, and their changes over time [1].

As an important lifestyle component, dietary data are being gathered at multiple time-points in the CLSA. Of interest were usual intakes of several key nutrients and foods of current concern in health promotion and chronic disease prevention in both younger and older adults (OA), and which have been the focus of population-based nutritional health promotion campaigns. They include intakes of total fat, and fatty acid classes (saturated, polyunsaturated, monounsaturated, omega-3 and *trans* fatty acids), as well as dietary fibre, calcium, vitamin D, and consumption of fruits and vegetables. Because of interview time constraints and the challenging logistics related to administration of a full food frequency questionnaire (FFQ), a brief instrument was sought for assessment of usual frequency of consumption of these items. Existing dietary screeners that addressed the foods and nutrients of interest were initially considered, specifically, the Block Dietary Screener [2], which ranks individuals with regard to their usual intakes of fat, fibre, calcium and vitamin D. Also of interest was the six-item Fruit and Vegetable Food Frequency Questionnaire developed by the Centers for Disease Control and Prevention (CDC) for the US Behavioral Risk Factor Surveillance System Survey (BRFSS [3]). The BRFSS questionnaire collects intake frequency, as occasions per day, week or month, of six categories of vegetables and fruit [4]. It has been used at the state level in the United States since 1990 [5] and was incorporated into the Canadian Community Health Survey by Health Canada [6]. The fruit and vegetable module has been validated against three 24-hour recalls (24HR) in a young adult population by trained dietitian interviewers, and results indicate that it can be used as a proxy for quantified intake in population groups [7].

However, because of constraints set by developers on instrument modification (e.g., changing the food list), as well as handling and cost considerations related to their use, it was decided to adapt a previously validated Canadian FFQ [8] as it best fit the needs of the CLSA and was specific to the population [9]. This approach also allowed us to conserve the BRFSS in the new tool. Furthermore, it limited the additional work needed for instrument modification, database preparation and data entry and analysis software as we had already successfully used the full FFQ [8] at recruitment into an ongoing cohort study on nutrition and healthy aging. The present paper describes the development, pretest and evaluation of the relative validity of the Short Diet Questionnaire (SDQ), developed to estimate usual consumption frequencies of fat, fibre, calcium and vitamin D, and fruit and vegetables.

## 2. Methods

### 2.1. Study Context

At the time of SDQ development, the CLSA was not yet in the field. Consequently, the SDQ was developed, pretested and validated in the “Quebec Longitudinal Study on Nutrition and Successful Aging” (“NuAge”), a cohort study that we were conducting at the time, and which is described in detail elsewhere [10]. Briefly, NuAge is a five-year observational study of 1793 community-dwelling men and women recruited in three age groups (70 ± 2 years; 75 ± 2 years; 80 ± 2 years), from a random sample of the Québec Health Insurance database (RAMQ) in the areas of Montréal, Laval, and Sherbrooke in Québec, Canada. Participants were cognitively and functionally intact and in good general health at recruitment, that is, having common manageable conditions such as hypertension or diabetes, but no serious illness limiting their continued participation. The study was approved by the Institutional Review Board at the Institut universitaire de gériatrie de Sherbrooke and the Institut universitaire de gériatrie de Montréal.

### 2.2. SDQ Development

A validated Canadian self-administered 78-item semi-quantitative FFQ [8] was used as the template for the SDQ. Items were extracted from the full FFQ to address the goals of the SDQ (see Table A1 in Appendix). The food list underwent several iterations to address issues related to content, food order, syntax and nomenclature, and participant burden. The six fruit and vegetable questions from the BRFSS were incorporated into the SDQ food list, keeping the exact BRFSS wording to permit comparison with existing Canadian data on fruit and vegetable intakes from other studies having used this module. Two formulations of the frequency component were considered: (1) frequency categories, where the respondent is presented with a series of predetermined options for frequency of intakes which requires that he/she choose the appropriate frequency choice, and (2) precise frequencies, where the respondent provides a specific number indicating the number of times the food item is consumed in one of the provided time periods: per day, per week, per month, or never/rarely. Portion size was not questioned.

Several versions of the instrument were examined internally before arriving at the pre-test versions of the SDQ, one with frequency categories and the other with precise frequencies, in both French and English. The full food list of the SDQ contains 30 food items and six beverage items (see Table A2 in in Appendix), as well as four additional questions on dietary habits relevant to the SDQ objectives. It queries usual consumption frequency in the previous 12 months of food sources of fats, fibre, calcium, vitamin D, regular and low-fat food choices, whole grains, calcium-fortified foods and beverages, and of a series of fruits and vegetables. For consistency with other national Canadian studies, the tool was designed to be interviewer-administered.

### 2.3. Pretest

The pretest of the SDQ took place at the Montreal study site in the summer of 2006. Research agents identified 30 participants from their roster who had agreed on their consent form to be re-contacted for additional studies, targeting approximately one-third in each of the NuAge age groups (70 years, 75 years, 80 years), evenly distributed among men and women, with a representative number of English-speaking NuAge participants to permit assessment of both French and English versions of the SDQ. The pretest was designed as a cross-over study, where half of the participants completed the “frequency categories” version first, followed by the “precise frequencies” version while the other half was assigned to the reverse order. The pretest SDQ was self-administered and returned by mail. Respondents entered their start and finish times on the SDQ to verify whether it could be completed in the allotted 15-minute timeframe. Structured, telephone-based cognitive interviews [11] were then carried out to assess difficulties with the test version of both frequency types of the SDQ, in both languages. Participants’ comments were used to clarify both language versions of the questionnaire following pretest. No quantitative analyses were carried out on the pretest questionnaires. A user’s manual was prepared to train the NuAge research agents in standardized administration of the SDQ and to provide them with answers and solutions to potential problems encountered in the use of the SDQ.

### 2.4. Validation Study

Recruitment into the SDQ validation study took place in 2007. NuAge participants were invited sequentially over an 8-month period to take part in the validation study without imposing additional selection criteria, but targeting equivalent proportions in each of the three age groups, both sexes and representing both languages. A total of 527 NuAge subjects took part in this study; 154 were completing T3 (NuAge year three) interviews and 373 were in their T4 wave of data collection. Current diet was assessed using three non-consecutive 24-hour diet recalls (24HR) collected at each annual interview in face-to-face and telephone interviews by a research dietitian using the USDA 5-step multiple pass method [12], and the 24HR was designated as the reference instrument. The 24HR interviews were carried out by professional research dietitians who adhered to a strict protocol. Since the CLSA Tracking Cohort was to be assembled by Statistics Canada which carries out all assessments by interview (in-person or by telephone), the SDQ was administered in telephone interviews by trained NuAge research dietitians, prior to collecting the second of three 24HR.

### 2.5. Data Handling and Dietary Analysis: Validation Study

Because the purpose of this study was to determine the validity of the SDQ, daily consumption frequencies of each line item were not tabulated separately, but were data-entered from SDQ responses into William™ customized data entry software (©Multispectra, 1997–2004) and output files were then imported directly into the SDQ data entry and analysis utility based on a Microsoft Access™ platform with a database and nutrient calculation algorithms adapted from the full FFQ [8]. Because portion size is not queried in the SDQ, it was imputed into the SDQ database as a standard (medium) portion in grams, from the NuAge study FFQ database to permit calculation of point estimates for comparison with the 24HR results. Nutrient analyses were thus done using the participants’ reported frequencies of consumption of each line item, with the adapted SDQ database based on the 2007b Canadian Nutrient File (CNF) [13] and results were output as daily nutrient and food intake estimates. The 24HR were analyzed using CANDAT nutrient analysis software (version 10, ©Godin London Inc., London, ON, Canada), based on the then-current 2001b CNF [13]. The means of the three 24HR were used in validation analyses, and software developed by our group (CalculateurGAC©) was run on the 24HR food codes and Canada’s Food Guide (CFG) subgroup codes to generate the four food groups of CFG in order to derive fruit and vegetable servings from the 24HR. Because the SDQ nutrient analysis targeted usual intakes of a limited set of nutrients and foods, the validation analyses were restricted to these target nutrients and foods from both the SDQ and the mean of the three non-consecutive 24HR.

### 2.6. Statistical Analyses

Estimated daily nutrient intakes for dietary fibre, calcium, vitamin D, total fat, cholesterol, saturated, monounsaturated, polyunsaturated, and *trans* fat, and servings of fruit and vegetables compiled from both instruments were examined using descriptive statistics to compute central tendencies. The test (SDQ) and reference (24HR) instruments were compared using paired t-tests for normally distributed data and Wilcoxon ranked non-parametric tests, and Spearman rank correlation analysis for data with skewed distributions. Joint classification of the targeted nutrient and food intake distributions from the SDQ and 24HR was assessed using cross-classification analyses, where participants were categorized into quartiles of consumption of nutrients and food group of interest, and the extent of exact and contiguous concordance, and frank misclassification was determined [14].

## 3. Results

Average completion time for both pretest versions of the SDQ was approximately 14 minutes (data not shown). The “precise frequencies” version of the pretest SDQ was retained in line with pretest participants’ comments and to ensure consistency with diet modules based on the BRFSS previously used by Statistics Canada and to allow for easier comparison with other studies using this mode of questioning. The SDQ validation study was carried out among 396 NuAge participants (54.8% female) who had complete data in both the test and reference instruments (Table 1). Most nutrient intakes and the number of servings of fruit and vegetables estimated from the SDQ were significantly lower than those estimated by the mean of three non-consecutive 24HR (*p* < 0.05), with the exception of calcium and vitamin D which were significantly higher compared to the 24HR, and saturated and trans fat (both NS) (Table 2). Spearman correlations between the SDQ and 24HR were low to moderate and statistically significant (*p* < 0.01), ranging from 0.19 (cholesterol: 27 mg/day lower than the 24HR) to 0.45 (fruits and vegetables: 1 portion/day lower than the 24HR) (Table 3). The arithmetic mean unadjusted Spearman correlation between the SDQ and the mean of three non-consecutive 24HR for the nine nutrients was 0.34, and it was 0.31 for the nine nutrients plus fruits and vegetables (data not shown). Finally, it can be seen in Table 4 that for all nutrients combined, 33.9% were jointly classified into identical quartiles of the distribution, 72.8% into identical and contiguous quartiles, and only 6.7% were frankly misclassified. Similar cross-classification results were observed in men and in women and there were no gender-related differences (data not shown).

**Table 1:** NuAge participants with complete data in both dietary data collection instruments, SDQ validation study (*n* = 396).

Sex	Age Group (years)			Total
	*n* (%)			
	67–72	73–77	78–84	
Male	65 (46.1)	46 (41.1)	68 (47.6)	179 (45.2)
Female	76 (53.9)	66 (58.9)	75 (52.4)	217 (54.8)
All	141 (35.6)	112 (28.3)	143 (36.1)	396 (100)

**Table 2:** Estimated intakes of nutrients of interest from SDQ and mean of three non-consecutive 24HR, SDQ validation study (*n* = 396).

Nutrient and Dietary Variables	Dietary Assessment Method				*p*-Value ^1^
	SDQ		Mean of Three Non-Consecutive 24HR		
	Mean	SD	Mean	SD	
Dietary fibre (g)	15.4	6.2	19.6	7.7	0.0001
Calcium (mg)	946	465	768	334	0.0001
Vitamin D (ug)	5.70	2.91	5.06	3.87	0.003/0.0001
Total fat (g)	63.7	25.5	69.5	26.2	0.0001
Cholesterol (mg)	229	86	256	132	0.0001/0.006
Saturated fat (g)	22.4	9.2	23.3	10.9	0.163/0.423
Monounsaturated fat (g)	24.5	10.5	25.9	10.9	0.04/0.053
Polyunsaturated fat (g)	11.4	5.0	13.6	5.7	0.0001
Trans fat (g)	0.7	0.4	0.8	0.9	0.03/0.829
Number of servings of fruit and vegetables ^2^	4.5	1.9	5.5	3.4	0.0001

^1^ paired *t*-test/Wilcoxon signed rank test; ^2^ *n* = 395.

**Table 3:** Associations between nutrient estimates from SDQ and mean of three non-consecutive 24HR, SDQ validation study (*n* = 396).

Nutrient and Dietary Variables	Spearman r ^1,2^
Dietary fibre (g)	0.34
Calcium (mg)	0.41
Vitamin D (ug)	0.33
Total fat (g)	0.26
Cholesterol (mg)	0.19
Saturated fat (g)	0.30
Monounsaturated fat (g)	0.28
Polyunsaturated fat (g)	0.22
Trans fat (g)	0.30
Number of servings of fruit and vegetables	0.45

^1^ All correlations significant (*p* ranged from <0.01 to <0.001); ^2^ Non-parametric correlations reported as variables did not follow a normal distribution.

**Table 4:** Cross-classification of nutrient estimates from SDQ and mean of three non-consecutive 24HR, SDQ validation study (*n* = 396).

Nutrient and Dietary Variables	% in Identical Quartile	% in Identical and Contiguous Quartile	% in Opposite Quartile ^1^
Dietary fibre (g)	36.4	75.8	6.8
Calcium (mg)	41.7	77.1	5.3
Vitamin D (ug)	34.1	73.5	6.1
Total fat (g)	29.5	71.4	7.6
Cholesterol (mg)	30.8	68.7	9.8
Saturated fat (g)	32.1	73.5	8.6
Monounsaturated fat (g)	37.1	69.9	6.8
Polyunsaturated fat (g)	29.8	68.9	6.1
Trans fat (g)	36.1	73.7	6.6
Number of servings of fruit and vegetables ^2^	32.2	77	3.5
Mean % classification (10 nutrients plus fruit and vegetables)	33.9	72.8	6.7

^1^ Frank misclassification (Q1:Q4); ^2^ *n* = 395.

## 4. Discussion

Brief dietary measurement instruments have been developed to assess intakes of single nutrients or foods such as fat, fruits and vegetables and to examine relationships between certain dietary exposures and risk of chronic disease. Many have been compared to other dietary assessment measures with known validity [15]. The present study reports on the relative validity of a 36-item frequency-based Short Diet Questionnaire compared to the mean of three non-consecutive, quantitative 24HR, developed for use in the population-based Canadian Longitudinal Study on Aging, compared to the mean of three non-consecutive, quantitative 24HR. While many brief instruments have focussed mainly on fat intakes or on fruit and vegetables [15,16], the SDQ was developed to estimate older adults’ usual consumption frequencies over a 12-month period of a set of key nutrients and foods that have been the focus of nutritional health promotion programmes targeting this segment of the population. Relative validation of the SDQ was carried out in a large sample (*n* = 396) compared to other studies of this type, and the reference instrument was collected rigourously. To our knowledge, this is the first study of its type to be conducted among community-dwelling older adults. Consequently, while comparisons with other studies in this population group were not possible, because these older adults were cognitively intact there is no basis for expecting less accurate reporting of their intakes on either the test or reference instruments. Although the results showed some inconsistencies, where certain nutrients were underestimated while others were overestimated by the SDQ relative to the 24HR, others have observed that brief instruments tend to overestimate fat and underestimate fruits and vegetables [17]. However, almost three-quarters of participants were cross-classified into the same section of the distribution by both test and reference instruments, providing evidence of the SDQ’s reasonable measurement properties.

While correlations between the key nutrients and foods estimated by the SDQ and the reference method (means of three non-consecutive 24HR) were quite modest, they were similar to those found in the literature for some of these variables. For example, a 43-item FFQ (Healthy Doc) administered by Spencer *et al.* [17] to 88 medical students estimated intakes of fruit and vegetables at 3.8 servings per day, only slightly lower than the 4.3 servings from the mean of five 24HR, with a Pearson correlation of 0.50. Using the 19-item NCI Fruit and Vegetable Screener (FVS) in an age and ethnically diverse sample of 590 adults, Greene *et al.* [18] obtained significant Pearson correlations ranging from 0.31 to 0.47 for men, and 0.43 to 0.63 for women between the FVS and multiple 24HR, depending on the sub-sample and version of their screener. Our results lined up closely to these findings, with 4.5 servings of fruits and vegetables per day estimated from the SDQ, compared to 5.5 servings daily from the three 24HR, and a significant, positive unadjusted Spearman rank correlation of 0.45.

Associations on fat from the SDQ and 24HR were also similar to those of Spencer *et al.* [17], with an unadjusted Spearman correlation of 0.26 between total fat estimated from the SDQ and the mean of the three 24HR, compared to the Spencer study which reported an adjusted, deattenuated Pearson correlation of 0.36 for fat between their brief FFQ and the mean of five 24HR.

Since the SDQ was not designed to assess the whole diet, we could not calculate energy intakes or estimate the percent of energy from fat. However, Thompson *et al.* [19] obtained a deattenuated Pearson correlation of 0.36 for percent energy from lipids from the 16-question NCI percentage of energy from fat short instrument (PFat) compared to multiple 24HR, suggesting that we can expect correlations between short diet questionnaires and quantitative, multiple 24HR to be in this modest range, similar to the present study.

It is difficult to contextualize results from this study with others, because of the heterogeneity of short dietary instruments and validation studies in the literature, including differing reference timeframes (for example the FVS asks for consumption frequencies over the last month), the use of implicit or explicit portion sizes in addition to frequency in some instruments [18], reporting on comparisons to “multiple” 24HR without specifying the number, and highly divergent samples and sample sizes. Although some studies have used a measurement error model to deattenuate correlations between the short dietary instrument and the reference measure, we have presented raw, unadjusted correlations from the SDQ and 24HR which fall into same range as adjusted correlations.

The study has limits. First, it must be acknowledged that all self-report dietary assessment methods are fraught with error. However, despite their age, the study participants were cognitively intact, which precludes expectation of poor results in this sample. Second, frequency-based dietary assessment instruments and quantitative tools such as 24HR call upon a different set of cognitive processes in order to respond to the food consumption questions. Consequently, respondents may have had difficulties with the notion of frequency on the SDQ, or could have forgotten to report some foods eaten on the 24HR assessment despite interviewer-prompts and cues on the 24HR, thus compounding errors and attenuating associations between the two instruments. Third, to permit calculation of point estimates for the validation analyses, SDQ portion sizes were imputed using medium portions from the parent NuAge FFQ nutrient database for all respondents, which may have induced a “regression to the mean” bias in comparing SDQ results to those from the 24HR, the “true” reference intakes. Fourth, the modest correlation coefficients could have been inflated due to the comparison of two potentially error-prone instruments, the SDQ and 24HR. Furthermore, this was a sub-study carried out within an ongoing cohort study, and certain participants reported confusion during the SDQ telephone interviews because the SDQ reminded them of the full FFQ that they had completed earlier in the study. In addition, based on comments noted by interviewers suggesting some uncertainty as to whether certain participants were aware that they had consumed fortified foods, the accuracy of their responses on consumption of omega-3 fatty acid or calcium-fortified foods may be questioned. Finally, because they had agreed to participate in the SDQ validation study, these respondents may have been particularly interested in diet, and thus not a representative sample.

## 5. Conclusions

The SDQ is a reasonably accurate, rapid, well-accepted approach for ranking usual consumption frequencies of selected nutrients and foods of interest. As such, it could serve in population health and chronic disease risk studies conducted among younger and older adults as a tool for rapid assessment of usual consumption frequencies of these foods and nutrients and to compare changes in patterns of consumption over time. Still, its limitations as a dietary assessment tool must be considered due to its focus only on selected nutrients and foods, as well as its qualitative nature which could result in underestimation of exposure to other dietary constituents. Consequently the SDQ would be inappropriate in studies where the objective is to obtain quantitative data on the whole diet. The first wave of administration of the SDQ to CLSA participants aged 45 to 85 years in the in-home interviews began in May 2012 and was completed in June 2015. Data entry is underway. Considering its intended use in the CLSA on multiple occasions over the 20-year follow-up, as well as the broader age range and more diverse population than the one that took part in the present relative validation study, further testing is necessary to determine its ability to accurately reflect consumption patterns of the target nutrients and foods considering different age groups, regional dietary variation and cognition in the older segment of the CLSA cohort. Additional testing of the SDQ against a full FFQ and a series of repeated 24HR would also permit further exploration of these issues, and allow for calibration of the SDQ to enhance its measurement properties.

## Appendix

**Table A1:** Sample food list modifications from the full FFQ to the SDQ.

Full FFQ ^1^	SDQ
High-fibre breakfast cereals (All Bran, 100% Bran, Bran Flakes, muesli…)	High-fibre breakfast cereals (All Bran, 100% Bran, Bran Flakes, muesli…)
2 categories:	Merged into 1 category:
▪ Bread: whole wheat, bran, multigrain, rye commercial sliced ▪ Other whole-wheat bread (crusty bread, hamburger/hot dog buns, tortillas, bagels, pitas…)	▪ Bread: whole wheat, bran, multigrain, rye (sliced, crusty, hamburger/hot dog buns, bagels, pita...)
2 categories:	Merged into 1 category:
▪ Beef (ground, hamburger, roast, steak, in cubes...)	▪ Beef, pork (ground, hamburger, roast, steak, in cubes...)
	Other meats (veal, lamb, game…) (ground, hamburger, roast, steak, in cubes...)
	Chicken, turkey
▪ Salmon, trout, sardines, herring, tuna	Addition of another species of fish: ▪ Salmon, trout, sardines, herring, tuna, mackerel
2 categories:	Merged into 1 category:
▪ Sausages, hot dogs ▪ Ham, cold cuts or smoked meats, bacon...	▪ Sausages, hot dogs, ham, cold cuts or smoked meats, bacon...

^1^ Shatenstein *et al.* [8]

**Table A2:** Short Diet Questionnaire food and beverage list.

Foods
High-fiber breakfast cereals (All Bran, 100% Bran, Bran Flakes, muesli…)
Whole-wheat breads, bran breads, multigrain breads, rye breads (sliced, crusty, hamburger bun, hot dog bun, bagel, pita, ...)
Beef, pork (ground, hamburgers, roast beef, steak, cubed…)
Other meats (veal, lamb, game…) (ground, hamburgers, roast, steak, cubed…)
Chicken, turkey
Salmon, trout, sardines, herring, tuna, mackerel (fresh, frozen or canned)
Sausages, hot dogs, ham, smoked meat, bacon...
Patés, cretons, terrines...
Sauces and gravies (brown, white, BBQ, …)
**Omega-3** eggs
All egg dishes except omega 3 eggs (eggs, omelette, quiche...)
Legumes: beans, peas, lentils
Nuts, seeds and peanut butter
Fruit (fresh, frozen, canned)
Green salad (lettuce, with or without other ingredients)
Potatoes (boiled, mashed or baked)
French fries or pan-fried potatoes, poutine
Carrots (fresh, frozen, canned, eaten on their own or with other food, cooked or raw)
Other vegetables (except carrots, potatoes or salad)
All **low-fat** cheeses
All **regular** cheeses
Yogurt (**low-fat**)
Yogurt (**regular**)
**Calcium-fortified** foods (soy pudding, ...)
Ice cream, ice milk, frozen yogurt, milk-based desserts (puddings, …)
Salty snacks (**regular** chips, crackers, ...)
Cakes, pies, doughnuts, pastries, cookies, muffins...
Chocolate bars
Butter or **regular** margarine on bread or on cooked vegetables **only**
**Regular** vinaigrettes, salad dressings, mayonnaise, homemade or commercial dips
**Beverages**
100% pure fruit juices (orange, grapefruit or tomato, ...)
**Calcium-fortified** juices
Whole milk 3.25% milk fat for drinking
2%, 1%, skim milk for drinking
**Calcium-fortified** milk (35% or more calcium)
Other **calcium-fortified** beverages (soy drink, ...)
